# EEG-Based Emotion Recognition: A State-of-the-Art Review of Current Trends and Opportunities

**DOI:** 10.1155/2020/8875426

**Published:** 2020-09-16

**Authors:** Nazmi Sofian Suhaimi, James Mountstephens, Jason Teo

**Affiliations:** Faculty of Computing & Informatics, Universiti Malaysia Sabah, Jalan UMS, Kota Kinabalu 88400, Sabah, Malaysia

## Abstract

Emotions are fundamental for human beings and play an important role in human cognition. Emotion is commonly associated with logical decision making, perception, human interaction, and to a certain extent, human intelligence itself. With the growing interest of the research community towards establishing some meaningful “emotional” interactions between humans and computers, the need for reliable and deployable solutions for the identification of human emotional states is required. Recent developments in using electroencephalography (EEG) for emotion recognition have garnered strong interest from the research community as the latest developments in consumer-grade wearable EEG solutions can provide a cheap, portable, and simple solution for identifying emotions. Since the last comprehensive review was conducted back from the years 2009 to 2016, this paper will update on the current progress of emotion recognition using EEG signals from 2016 to 2019. The focus on this state-of-the-art review focuses on the elements of emotion stimuli type and presentation approach, study size, EEG hardware, machine learning classifiers, and classification approach. From this state-of-the-art review, we suggest several future research opportunities including proposing a different approach in presenting the stimuli in the form of virtual reality (VR). To this end, an additional section devoted specifically to reviewing only VR studies within this research domain is presented as the motivation for this proposed new approach using VR as the stimuli presentation device. This review paper is intended to be useful for the research community working on emotion recognition using EEG signals as well as for those who are venturing into this field of research.

## 1. Introduction

Although human emotional experience plays a central part in our daily lives, our scientific knowledge relating to the human emotions is still very limited. The progress for affective sciences is crucial for the development of human psychology for the benefit and application of the society. When machines are integrated into the system to help recognize these emotions, it would improve productivity and reduce the cost of expenditure in many ways [1], for example, integrations of machines into the society such as education where observations of student's mental state towards the contents of the teaching materials being engaging or nonengaging can be detected. Medical doctors would be able to assess their patients' mental conditions and provide better constructive feedback to improve their health conditions. The military will be able to train their trainees in simulated environments with the ability to assess their trainees' mental conditions in combat situations.

A person's emotional state may become apparent through subjective experiences, internal and external expressions. Self-evaluation reports such as the Self-Assessment Manikin (SAM) [2] is commonly used for evaluating the mental state of a person by measuring the three independent and bipolar dimensions [3], presented visually to the person by reflecting images of pleasure-displeasure, degree of arousal, and dominance-submissiveness. This method provides an alternative to the sometimes more difficult assessment of psychological evaluations of a patient done by a medical profession where they would require thorough training and experience to understand the patient's mental health conditions. However, the validity and corroboration of the information provided from the patient using the SAM report are unreliable given that many people have difficulty expressing themselves honestly or lack of knowledge or grasp towards their mental state. SAM is also not feasible to be conducted on young children or elders due to the limitation of literacy skills [4]. Therefore, the physiological signals that are transported throughout the human body can provide health information directly from patients to medical professionals and evaluate their conditions almost immediately. The brainwave signal of a human being produces insurmountable levels of neuron signals that manage all functionalities of the body. The human brain stores the emotional experiences that are gathered throughout their lifetime. By tapping directly into the brainwave signals, we can examine the emotional responses of a person when exposed to certain environments. With this information provided from the brainwave signals, it can help strengthen and justify the person is physically fit or may be suffering from mental illness.

The architectural design and cost of the EEG headset differ differently. The difference here is that the type of electrodes used to collect the brainwave signals affects the quality as well as the duration of setup [5–7]. There are also a different number of electrodes placed across the human scalp, and the resolution of these EEG headsets differs depending on the build quality and technological accessibility [8–10]. Due to the sensitivity of the electrodes, many users are required to be very static when the brainwave collection procedure is initiated, and any small body or head movements may accidentally detach the electrodes out from the scalp and require to be reattached to the head which could waste time and materials. Any hair strands where the electrodes would be placed had to be removed to receive proper connection of the brainwave signals. Therefore, people with large hair volumes would face difficulty as the hair would need to be shifted or removed. Artefacts are noises produced from muscle movements such as eye blinking, jaw clenching, and muscle twitches which would be picked up by the electrodes [11–14]. Furthermore, external interferences such as audio noise or sense of touch may also introduce artefacts into the brainwave signals during collection, and these artefacts will need to be removed by the use of filtering algorithms [15–20]. Finally, the brainwave signals will need to be transformed from time domain to frequency domain using fast Fourier transform (FFT) [21] to assess and evaluate the specific brainwave bands for emotion recognition with machine learning algorithms.

Since the last comprehensive review for emotion recognition was published by Alarcao and Fonseca [22], this review paper will serve as an update on the previously reviewed paper. The paper is organized as follows: Section 2 includes the methodology of reviewing this paper by using specific keywords search.  Section 3 will cover the definition of what emotion is, EEG, brainwave bands, general positions of EEG electrodes, comparison between clinical and low-cost wearable EEG headset, emotions in the brain, and virtual reality (VR).  Section 4 will review past studies of emotion classification by comparing the types of stimulus, emotion classes, dataset availability, common EEG headset used for emotion recognition, common algorithms and performances of machine learning in emotion recognition, and participants involved.  Section 5 provides discussion, and finally, Section 6 concludes the study.

## 2. Methodology

The approach adopted in this state-of-the-art review firstly performs queries on the three most commonly accessed scholarly search engine and database, namely, Google Scholar, IEEE Explore, and ScienceDirect, to collect papers for the review using the keywords *“Electroencephalography” or “EEG”* + “*Emotion”* + *“Recognition”* or *“Classification”* or *“Detection”* with the publication year ranging only from 2016 to 2019. The papers resulting from this search are then carefully vetted and reviewed so that works that were similar and incremental from the same author were removed, leaving only distinctly significant novel contributions to EEG-based emotion recognition.

### 2.1. State of the Art

In the following paragraphs, the paper will introduce the definitions and representations of emotions as well as some characteristics of the EEG signals to give some background context for the reader to understand the field of EEG-based emotion recognition.

## 3. Emotions

Affective neuroscience is aimed to elucidate the neural networks underlying the emotional processes and their consequences on physiology, cognition, and behavior [23–25]. The field has been historically centered around defining the universal human emotions and their somatic markers [26], clarifying the cause of the emotional process and determining the role of the body and interoception in feelings and emotions [27]. In affective neuroscience, the concept of emotions can be differentiated from various constructs such as feelings, moods, and affects. Feelings can be viewed as a personal experience that associates itself with that emotion. Moods are diffuse affective states that generally last longer than emotions and are less intense than emotions. Lastly, affect is an encompassing term that describes the topics of emotions, feelings, and moods altogether [22].

Emotions play an adaptive, social, or motivational role in the life of human beings as they produce different characteristics indicative of human behavior [28]. Emotions affect decision making, perception, human interactions, and human intelligence. It also affects the status of humans physiologically and psychologically [29]. Emotions can be expressed through positive and negative representations, and from them, it can affect human health as well as work efficiency [30].

Three components influence the psychological behavior of a human, which are personal experiences, physiological response, and behavioral or expressive response [31, 32]. Emotions can be described as being responsive to discrete or consistent responses of events with significance for the organisms [33] which are brief in duration and corresponds to a coordinated set of responses.

To better grasp the kinds of emotions that are being expressed daily, these emotions can be viewed from categorical perspective or dimensional perspective. The categorical perspective revolves around the idea of basic emotions that have been imprinted in our human physiology. Ekman [34] states that there are certain characteristics of basic emotions: (1) humans are born with emotions that are not learned; (2) humans exhibit the same emotions in the same situation; (3) humans express these emotions in a similar way; and (4) humans show similar physiological patterns when expressing the same emotions. Through these characteristics, Ekman was able to summarize the six basic emotions of happiness, sadness, anger, fear, surprise, and disgust, and he viewed the rest of the emotions as a byproduct of reactions and combinations of the basic emotions. Plutchik [35] proposes that there are eight basic emotions described in a wheel model, which are joy, trust, fear, surprise, sadness, disgust, anger, and anticipation. Izard (Izard, 2007; Izard, 2009) describes that (1) basic emotions were formed in the course of human evolution and (2) each basic emotion corresponded to a simple brain circuit and there was no complex cognitive component involved. He then proposed his ten basic emotions: interest, joy, surprise, sadness, fear, shyness, guilt, anger, disgust, and contempt. On the other hand, from the dimensionality perspective, the emotions are mapped into valence, arousal, and dominance. Valence is measured from positive to negative feelings, arousal is measured from high to low, and similarly, dominance is measured from high to low [38, 39].

Understanding emotional signals in everyday life environments becomes an important aspect that influences people's communication through verbal and nonverbal behavior [40]. One such example of emotional signals is expressed through facial expression which is known to be one of the most immediate means of human beings to communicate their emotions and intentions [41]. With the advancement of technologies in brain-computer interface and neuroimaging, it is now feasible to capture the brainwave signals nonintrusively and to measure or control the motions of devices virtually [42] or physically such as wheelchairs [43], mobile phone interfacing [44], or prosthetic arms [45, 46] with the use of a wearable EEG headset. Currently, the advancement of artificial intelligence and machine learning is being actively developed and researched to adopt to newer applications. Such applications include neuroinformatics field which studies the emotion classification by collecting brainwave signals and classifying them using machine learning algorithms. This would help improve human-computer interactions to meet human needs [47].

### 3.1. The Importance of EEG for Use in Emotion Classification

**Figure 1:**
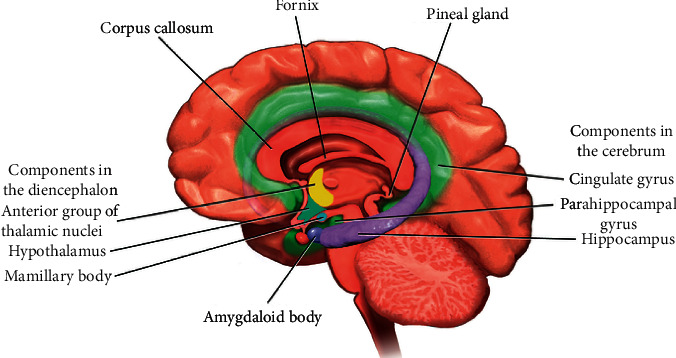
The limbic system (source: https://courses.lumenlearning.com/boundless-psychology/chapter/biology-of-emotion/#:∼:text=The%20limbic%20system%20is%20the,thalamus%2C%20amygdala%2C%20and%20hippocampus).

EEG is considered a physiological clue in which electrical activities of the neural cells cluster across the human cerebral cortex. EEG is used to record such activities and is reliable for emotion recognition due to its relatively objective evaluation of emotion compared to nonphysiological clues (facial expression, gesture, etc.) [48, 49]. Works describing that EEG contains the most comprehensive features such as the power spectral bands can be utilized for basic emotion classifications [50]. There are three structures in the limbic system as shown in Figure 1, where the brain heavily implicates emotion and memory: the hypothalamus, amygdala, and hippocampus. The hypothalamus handles the emotional reaction while the amygdala handles external stimuli that process the emotional information from the recognition of situations as well as analysis of potential threats. Studies have suggested that amygdala is the biological basis of emotions that store fear and anxiety [51–53]. Finally, the hippocampus integrates emotional experience with cognition.

### 3.2. Electrode Positions for EEG

**Figure 2:**
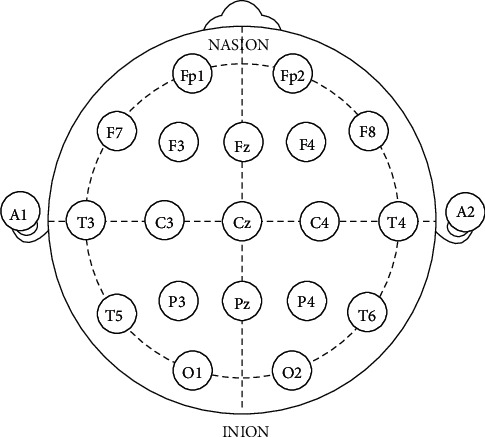
The 10–20 EEG electrode positioning system (source: [56]).

To be able to replicate and record the EEG readings, there is a standardized procedure for the placements of these electrodes across the skull, and these electrode placement procedures usually conform to the standard of the 10–20 international system [54, 55]. The “10 and “20” refers to the actual distances between the adjacent electrodes either 10% or 20% of the total front to back or right to the left of the skull. Additional electrodes can be placed on any of the existing empty locations.  Figure 2 shows the electrode positions placed according to the 10–20 international system.

**Figure 3:**
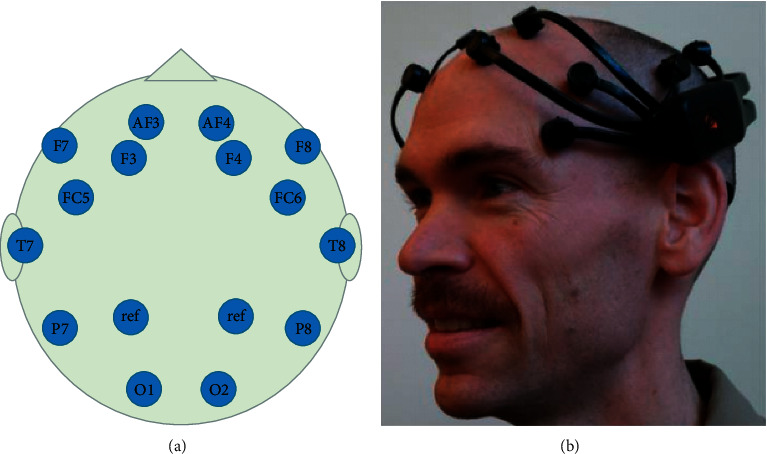
A 14-channel low-cost wearable EEG headset Emotiv EPOC worn by subject (source: [57]).

**Figure 4:**
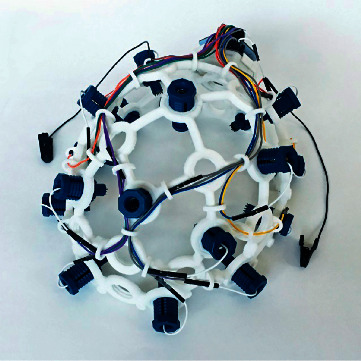
8- to 16-channel Ultracortex Mark IV (source: https://docs.openbci.com/docs/04AddOns/01-Headwear/MarkIV).

Depending on the architectural design of the EEG headset, the positions of the EEG electrodes may differ slightly than the standard 10–20 international standard. However, these low-cost EEG headsets will usually have electrodes positioned at the frontal lobe as can be seen from Figures 3 and 4. EEG headsets with a higher number of channels will then add electrodes to the temporal, parietal, and occipital lobe such as the 14-channel Emotiv EPOC+ and Ultracortex Mark IV. Both these EEG headsets have wireless capabilities for data transmission and therefore have no lengthy wires dangling around their body which makes it feasible for this device to be portable and easy to setup. Furthermore, companies such as OpenBCI provide 3D-printable designs and hardware configurations for their EEG headset which provides unlimited customization to their headset configurations.

### 3.3. Clinical-Grade EEG Headset vs. Wearable Low-Cost EEG Headset

Previously, invasive electrodes were used to record brain signals by penetrating through the skin and into the brain, but technology improvements have made it possible for electrical activity of the brain to be recorded by using noninvasive electrodes placed along the scalp of the brain. EEG devices focus on event-related (stimulus onset) potentials or spectral content (neural oscillations) of EEG. They can be used to diagnose epilepsy, sleep disorders, encephalopathies (brain damage or malfunction), and other brain disorders such as brain death, stroke, or brain tumors. EEG diagnostics can help doctors to identify medical conditions and appropriate injury treatments to mitigate long-term effects.

EEG has advantages over other techniques because of the ease to provide immediate medical care in high traffic hospitals with lower hardware costs as compared to magnetoencephalography. In addition, EEG does not aggravate claustrophobia in patients, can be used for patients who cannot respond, or cannot make a motor respond or attending to a stimulus where EEG can elucidate stages of processing instead of just final end results.

**Figure 5:**
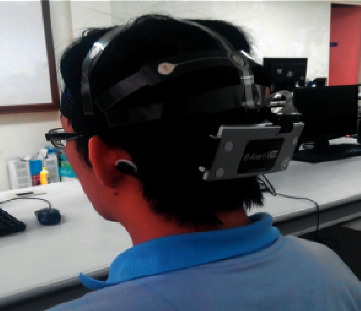
A medical-grade EEG headset B-Alert X10, 10 channels (source: [59]).

tMedical-grade EEG devices would have channels ranging between 16 and 32 channels on a single headset or more depending on the manufacturer [58] and it has amplifier modules connected to the electrodes to amplify these brainwave signals which can be seen in Figure 5. The EEG devices that are used in clinics help to diagnose and characterize any symptoms obtained from the patient and these data are then interpreted by a registered medical officer for medical interventions [60, 61]. A study conducted by Obeid and Picone [62] where the clinical EEG data stored in secure archives are collected and made publicly available. This would also help establish a best practice for curation and publication of clinical signal data.  Table 1 shows the current EEG market and the pricing of its products available for purchase. However, the cost of EEG headsets is not disclosed from the middle-cost range most likely due to the sensitivity of the market price or they would require clients to specifically order according to their specifications unlike the low-cost EEG headsets, which disclosed the cost of their EEG headsets.

**Figure 6:**
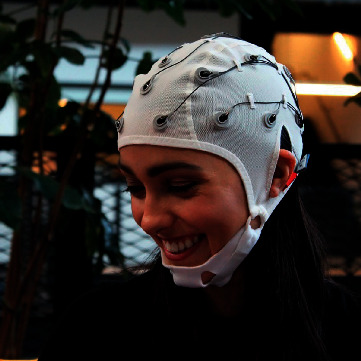
21-channel OpenBCI electrode cap kit (source: https://docs.openbci.com/docs/04AddOns/01-Headwear/ElectrodeCap).

A low-cost, consumer-grade wearable EEG device would have channels ranging from 2 to 14 channels [58]. As seen from Figure 6, the ease of setup while wearing a low-cost, consumer-grade wearable EEG headset provides comfort and reduces the complexity of setting up the device on the user's scalp, which is important for both researchers and users [63]. Even with the lower performance of wearable low-cost EEG devices, it is much more affordable compared to the standard clinical-grade EEG amplifiers [64]. Interestingly, the supposedly lower performance EEG headset could outperform a medical-grade EEG system with a lesser number of electrodes [65]. The lower cost of wearable EEG systems could also detect artefacts such as eye blinking, jaw clenches, muscle movements, and power supply line noises which can be filtered out during preprocessing [66]. The brain activity of the wireless portable EEG headset can also assist through the imagined directional inputs or hand movements from a user, which was compared and shown to perform better than medical-grade EEG headsets [67–70].

### 3.4. Emotions in the Brain

In recent developments, a high number of neurophysiological studies have reported that there are correlations between EEG signals and emotions. The two main areas of the brain that are correlated with emotional activity are the amygdala and the frontal lobe. Studies showed that the frontal scalp seems to store more emotional activation compared to other regions of the brain such as temporal, parietal, and occipital [71].

In a study regarding music video excerpts, it was observed that higher frequency bands such as gamma were detected more prominently when subjects were listening to unfamiliar songs [72]. Other studies have observed that high-frequency bands such as alpha, beta, and gamma are more effective for classifying emotions in both valence and arousal dimensions [71, 73] (Table 2).

Previous studies have suggested that men and women process emotional stimuli differently, suggesting that men evaluate current emotional experiences relying on the recall of past emotional experiences, whereas women seemed to directly engage with the present and immediate stimuli to evaluate current emotional experiences more readily [74]. There is also some evidence that women share more similar EEG patterns among them when emotions are evoked, while men have more individual differences among their EEG patterns [75].

In summary, the frontal and parietal lobes seem to store the most information about emotional states, while alpha, gamma, and beta waves appear to be most discriminative.

### 3.5. What Is Virtual Reality (VR)?

VR is an emerging technology that is capable of creating some amazingly realistic environments and is able to reproduce and capture real-life scenarios. With great accessibility and flexibility, the adaptation of this technology for different industries is limitless. For instance, the use of a VR as a platform to train fresh graduates to be better in soft skills while applying for a job interview can better prepare them for real-life situations [76]. There are also applications where moods can be tracked based on their emotional levels while viewing movies, thus creating a list of databases for movie recommendations for users [77]. It is also possible to improve social skills for children with autism spectrum disorder (ASD) using virtual reality [78]. To track all of the emotion responses of each person, the use of a low-cost wearable EEG that is wireless is now feasible to record the brainwave signals and then evaluate the mental state of the person with the acquired signals.

VR is used by many different people with many meanings. Some of the people would refer to this technology as a collection of different devices which are a head-mounted device (HMD), glove input device, and audio [79]. The first idea of a virtual world was presented by Ivan Sutherland in 1965 which he was quoted as saying: “make that (virtual) world in the window look real, sound real, feel real and respond realistically to the viewer's actions” [80]. Afterward, the first VR hardware was realized with the very first HMD with appropriate head tracking and has a stereo view that is updated correctly according to the user's head position and orientation [81].

From a study conducted by Milgram and Kishimo [82] regarding mixed reality, it is a convergence of interaction between the real world and the virtual world. The term mixed reality is also used interchangeably with augmented reality (AR) but most commonly referred to as AR nowadays. To further understand what AR really is, it is the incorporation of virtual computer graphic objects into a real three-dimensional scene, or alternatively the inclusions of real-world environment elements into a virtual environment [83]. The rise of personal mobile devices [84] especially in 2010 accelerated the growth of AR applications in many areas such as tourism, medicine, industry, and educations. The inclusion of this technology has been nothing short of positive responses [84–87].

In VR technology, the technology itself opens up to many new possibilities for innovations in areas such as healthcare [88], military [89, 90], and education [91].

## 4. Examining Previous Studies

In the following section, the papers obtained between 2016 and 2019 will be analyzed and categorized according to the findings in tables. Each of the findings will be discussed thoroughly by comparing the stimulus types presented, elapsed time of stimulus presentation, classes of emotions used for assessments, frequency of usage, the types of wearable EEG headsets used for brainwave collections and its costs, the popularity usage of machine learning algorithms, comparison of intra- and intersubject variability assessments, and the number of participants conducted in the emotional classification experiments.

### 4.1. Examining the Stimulus Presented

Recent papers collected from the years 2016 to 2019 found that the common approach towards stimulating user's emotional experience was music, music video, pictures, video clips, and VR. Of the five stimuli, VR (31.03%) was seen to have the highest common usage for emotion classification followed by music (24.14%), music videos and video clips (both at 20.69%), and pictures (3.45%) which can be observed in Table 3.

The datasets the researchers used to collect for their stimulation contents are ranked as follows: first is Self-Designed at 43.75%, second is DEAP at 18.75%, third are SEED, AVRS, and IAPS at 6.25%, and lastly, IADS, DREAMER, MediaEval, Quran Verse, DECAF, and NAPS all at 3.13%. The most prominent use for music stimuli all comes from the DEAP dataset [121] which is highly regarded and commonly referred to for its open access for researchers to conduct their research studies. While IADS [122] and MediaEval [123] are both open-source content for their music database with labeled emotions, it does not seem that researchers have utilized the database much or might be unaware of the availability of these datasets. As for video-related contents, SEED [124–126], DREAMER [127], and ASCERTAIN [107] do provide their video database either openly or upon request. Researchers who designed their own stimulus database used two different stimuli, which are music and video clips, and of those two stimuli approaches, self-designed with music stimuli have 42.86% and self-designed video clips have 57.14%. Table 3 provides the information for accessing the mentioned databases available for public usage.

One of the studies was not included in the clip length averaging (247.55 seconds) as this paper reported the total length instead of per clip video length. The rest of the papers in Table 4 have explicitly mentioned per clip length or the range of the video length (taken at maximum length) that were used to average out the length per clip presented to the participants. Looking into the length of the clips whether it is in pictures, music, video clips, or virtual reality when measured on average, the length per clip was 107 seconds with the shortest length at 15 seconds (picture) while the longest was at 820 seconds (video clip). This may not reflect properly with the calculated average length of the clip since some of the lengthier videos were only presented in one paper and again because DEAP was referred repeatedly (60 seconds).

Looking into VR focused stimuli, the researchers designed their own stimuli database that would fit into their VR environment since there is a lack of available datasets as those currently available datasets were designed for viewing from a monitor's perspectives. Affective Virtual Reality System (AVRS) is a new database designed by Zhang et al. [114] which combines IAPS [128], IADS, and China Affective Video System (CAVS) to produce a virtual environment that would accommodate VR headset for emotion classification. However, the dataset has only been evaluated using Self-Assessment Manikin (SAM) to evaluate the effectiveness of the AVRS system delivery of emotion and currently is still not made available for public access. Nencki Affective Picture System (NAPS) developed by Marchewka et al. [129] uses high-quality and realistic picture databases to induce emotional states.

### 4.2. Emotion Classes Used for Classification

30 papers studying emotion classification were identified, and only 29 of these papers are tabulated in Table 4 for reference on its stimuli presented, the types of emotions assessed, length of their stimulus, and the type of dataset utilized for their stimuli presentation to their test participants. Only 18 studies have reported the emotional tags used for emotion classification and the remaining 11 papers use the two-dimensional emotional space while one of the papers did not report the emotional classes used but is based on the DEAP dataset, and as such, this paper was excluded from Table 4. Among the 18 investigations that reported their emotional tags, an average number of 4.3 emotion classes were utilized and ranged from one to nine classes that were used for emotion classifications. There were a total of 73 emotional tags used for these emotional classes with some of the commonly used emotional classes such happy (16.44%), sad (13.70%), and fear (12.33%), which Ekman [34] has described in his six basic emotions research, but the other three emotion classes such as angry (5.48%), surprise (1.37%), and disgust (5.48%) were not among the more commonly used tags for emotional classifications. The rest of the emotional classes (afraid, amusement, anger, anguish, boredom, calm, contentment, depression, distress, empathy engagement, enjoyment, exciting, exuberance, frightened, frustration, horror, nervous, peaceful, pleasant, pleased, rage, relaxation, tenderness, workload, among others) were used only between 1.37% and 5.48% and these do not include valence, arousal, dominance, and liking indications.

Emotional assessment using nonspecific classes such as valence, arousal dominance, liking, positive, negative, and neutral had been used 28 times in total. Emotional assessment using the two-dimensional space such as valence and arousal where valence was used to measure the positive or negative emotions showed about 32.14% usage in the experiment and arousal where the user's level of engagement (passive or active) was also seen to have 32.14% usage in these papers. The lesser evaluated three-dimensional space where dominance was included showed only 7.14% usage. This may be due to the higher complexity of the emotional state of the user and requires them to have a knowledgeable understanding of their mental state control. As for the remainder nonspecific tags such as positive, negative, neutral, liking, these usages range between 3.57% and 10.71% only.

Finally, there were four types of stimuli used to evoke emotions in their test participants consisting solely of music, music videos, video clips, and virtual reality with one report that combines both music and pictures together. Music contains audible sounds that can be heard daily such as rain, writing, laughter, or barking as done from using IAPS stimulus database while other auditory sounds used musical excerpts collected from online musical repositories to induce emotions. Music videos are a combination of rhythmic songs with videos with dancing movements. Video clips pertaining to Hollywood movie segments (DECAF) or Chinese movie films (SEED) were collected and stitched according to their intended emotion representation needed to entice their test participants. Virtual reality utilizes the capability of being immersed in a virtual reality environment with users being capable of freely viewing its surroundings. Some virtual reality environments were captured using horror films or a scene where users are only able to view objects from its static position with environments changing its colours and patterns to arouse the users' emotions. The stimuli used for emotion classification were virtual reality stimuli having seen a 31.03% usage, music at 24.14%, both music videos and video clips at 20.69% usage, and finally the combination of music and picture at 3.45% single usage.

### 4.3. Common EEG Headset Used for Recordings

The tabulated information on the common usage of wearable EEG headsets is described in Table 5. There were 6 EEG recording devices that were utilized for EEG recordings. These headsets are NeuroSky, Emotiv EPOC+, B-Alert X10, Ag Electrodes, actiChamp, and Muse. Each of these EEG recording devices is ranked according to their usages: BioSemi ActiveTwo (40.00%), Emotiv EPOC+, and NeuroSky MindWave (13.33%), while the remainder had 6.67% usage from actiChamp, Ag/AgCK Sintered Ring Electrodes, AgCl Electrode Cap, B-Alert X10, and Muse. Among the six EEG recording devices here, only the Ag Electrodes are required to manually place its electrodes on the scalp of their subjects while the remaining five EEG recording devices are headsets that have preset electrode positions for researchers to place the headset easily over their subject's head. To obtain better readings from the electrodes of these devices, the Emotiv EPOC+ and Ag Electrodes are supplied with an adhesive gel to improve the signal acquisition quality from their electrodes and Muse only required to use a wet cloth applied onto the skin to improve their signal quality due to its dry electrode technology while the other three devices (B-Alert X10, actiChamp, and NeuroSky) do not provide recommendations if there is any need to apply any adhesive element to help improve their signal acquisition quality. All of these devices are capable of collecting brainwave frequencies such as delta, theta, alpha, beta, and gamma, which also indicates that the specific functions of the brainwave can be analyzed in a deeper manner especially for emotion classification, particularly based on the frontal and temporal regions that process emotional experiences. With regard to the regions of the brain, Emotiv EPOC+ electrode positions can be placed at the frontal, temporal, parietal, and occipital regions, B-Alert X10 and actiChamp place their electrode positions at the frontal and parietal region, Muse places their electrode positions at the frontal and temporal region, and NeuroSky places their electrode positions only at the frontal region. Ag Electrodes have no limitations on the number of electrodes provided as this solely depends on the researcher and the EEG recording device only.

Based on Table 5, of the 15 research papers which disclosed their headsets used, only 11 reported on their collected EEG brainwave bands with 9 of the papers having collected all of the five bands (delta, theta, alpha, beta, and gamma) while 2 of the papers did not collect delta band and 1 paper did not collect delta, theta, and gamma bands. This suggests that emotion classification studies, both lower frequency bands (delta and theta) and higher frequency bands (alpha, beta, and Gamma) are equally important to study and are the preferred choice of brainwave feature acquisition among researchers.

### 4.4. Popular Algorithms Used for Emotion Classification

The recent developments on human-computer interaction (HCI) that allows the computer to recognize the emotional state of the user provide an integrated interaction between human and computers. This platform propels the technology forward and creates vast opportunities for applications to be applied in many different fields such as education, healthcare, and military applications [131]. Human emotions can be recognized through various means such as gestures, facial recognition, physiological signals, and neuroimaging.

According to previous researchers, over the last decade of research on emotion recognition using physiological signals, many have deployed numerous methods of classifiers to classify the different types of emotional states [132]. Features such as K-nearest neighbor (KNN) [133, 134], regression tree, Bayesian networks, support vector machines (SVM) [133, 135], canonical correlation analysis (CCA) [136], artificial neural network (ANN) [137], linear discriminant analysis (LDA) [138], and Marquardt backpropagation (MBP) [139] were used by researchers to classify the different emotions. However, the use of these different classifiers makes it difficult for systems to port to different training and testing datasets, which generate different learning features depending on the way the emotion stimulations are presented for the user.

Observations were made over the recent developments of emotion classifications between the years 2016 and 2019 and it shows that many techniques described earlier were applied onto them with some other additional augmentation techniques implemented.  Table 6 shows the classifiers used and the performance achieved from these classifications, and each of the classifiers is ranked accordingly by popularity: SVM (31.48%), KNN (11.11%), NB (7.41%), MLP, RF, and CNN (5.56% each), Fisherface (3.70%), BP, Bayes, DGCNN, ELM, FKNN, GP, GBDT, Haar, IB, LDA, LFSM, neural network, neuro-fuzzy network, WPDAI-ICA, and HC (1.85% each) while one other used Biotrace+ (1.85%) software to evaluate their classification performance and it was unclear as to which algorithm technique was actually applied for the performance obtained.

As can be seen here, SVM and KNN were among the more popular methods for emotion classification and the highest achieved performance was 97.33% (SVM) and 98.37% (KNN). However, there were other algorithms used for emotion classification that performed very successfully as well and some of these classifiers which crossed the 90% margin were CNN (97.69%), DGCNN (90.40%), Fisherface (91.00%), LFSM (92.23%), and RF (98.20%). This suggests that other classification techniques may be able to achieve good performance or improve the results of the classification. These performances only show the highest performing indicators and do not actually reflect the general emotion consensus as some of these algorithms worked well on the generalized arousal and/or valence dimensions and in other cases used very specific emotional tags, and therefore, it is difficult to directly compare the actual classification performance across all the different classifiers.

### 4.5. Inter- and Intrasubject Classification in the Study of Emotion Classification

The definition of intersubject variability is the differences in brain anatomy and functionality across different individuals whereas intrasubject variability is the difference in brain anatomy and functionality within an individual. Additionally, intrasubject classification conducts classification using the training and testing data from only the same individual whereas intersubject classification conducts classification using training and testing data that is not limited to only from the same individual but from across many different individuals. This means that in intersubject classification, testing can be done without retraining the classifier for the individual being tested. This is clearly a more challenging task where the classifier is trained and tested using different individuals' EEG data. In recent studies, there has been an increasing number of studies that focused on appreciating rather than ignoring classification. Through the lens of variability, it could gain insight on the individual differences and cross-session variations, facilitating precision functional brain mapping and decoding based on individual variability and similarity. The application of neurophysiological biometrics relies on the intersubject variability and intrasubject variability where questions regarding how intersubject and intrasubject variability can be observed, analyzed, and modeled. This would entail questions of what differences could researchers gain from observing the variability and how to deal with the variability in neuroimaging. From the 30 papers identified, 28 indicated whether they conducted intrasubject, intersubject, or both types of classification.

The nonstationary EEG correlates of emotional responses that exist between individuals, namely, intersubject variability would be affected by the intrinsic differences in personality, culture, gender, educational background, and living environment, and individuals may have distinct behavioral and/or neurophysiological responses even when perceiving the same event. Thus, each individual is not likely to share the common EEG distributions that correlate to the same emotional states. Researchers have highlighted the significant challenges posed by intersubject classification in affective computing [140, 142–147]. Lin describes that for a subject-dependent exercise (intersubject classification) to work well, the class distributions between individuals have to be similar to some extent. However, individuals in real life may have different behavioral or physiological responses towards the same stimuli. Subject-independent (intrasubject classification) was argued and shown to be the preferable emotion classification approach by Rinderknecht et al. [148]. Nonetheless, the difficulty here is to develop and fit a generalized classifier that will work well for all individuals, which currently remains a grand challenge in this research domain.

From Table 6, it can be observed that not all of the researchers indicated their method of classifying their subject matter. Typically, setup descriptions that include subject-independent and across subjects refer to inter-subject classification while subject-dependent and within subjects refer to intra-subject classification. These descriptors were used interchangeably by researchers as there are no specific guidelines as to how these words should be used specifically in the description of the setups of these emotion classification experiments. Therefore, according to these descriptors, the table helps to summarize these papers in a more objective manner. From the 30 papers identified, only 18 (5 on intrasubject and 13 on intersubject) of the papers have specifically mentioned their classifications on the subject matter. Of these, the best performing classifier for intrasubject classification was achieved by RF (98.20%) by Kumaran et al. [93] on music stimuli while the best for intersubject classification was achieved by DGCNN (90.40%) by Song et al. [110] using video stimulations from SEED and DREAMER datasets. As for VR stimuli, only Hidaka et al. [116] performed using SVM (81.33%) but using only five subjects to evaluate its performance, which is considered to be very low when the number of subjects at minimal is expected to be 30 to be justifiable as mentioned by Alarcao and Fonseca [22].

### 4.6. Participants

From the 30 papers identified, only 26 of the papers have reported the number of participants used for emotion classification analysis as summarized in Table 7, and the table is arranged from the highest total number of participants to the lowest. The number of participants varies between the ranges from 5 to 100 participants, and 23 reports stated their gender population with the number of males (408) being higher than females (342) overall, while another 3 reports only stated the number of participants without stating the gender population. 7.70% was reported using less than 10 subjects, 46.15% reported using between 10 and 30 participants, and 46.15% reported using more than 30 participants.

16 reports stated their mean age groups ranging between 15.29 and 30 with an exception that there was a study on ASD (autism spectrum disorder) group being the youngest with the mean age of 15.29. Another 4 only reported their participants' age ranging between 18 and 28 [106, 120, 141, 150] while 2 other studies only reported they had volunteers from their university students [98, 115] and 1 other report stated they had 2 additional institutions volunteered in addition to their own university students [118].

The 2 reported studies with less than 10 participants [92, 119] have had their justifications on why they would be conducting with these numbers such that Horvat expressed their interest in investigating the stability of affective EEG features by running multiple sessions on single subjects compared to running large number of subjects such as DEAP with single EEG recording session for each subject. Lan was conducting a pilot study on the combination of VR using NAPS database with the Emotiv EPOC+ headset to investigate the effectiveness of both devices and later found that in order to achieve a better immersion experience, some elements of ergonomics on both devices have to be sacrificed.

The participants who volunteered to join for these experiments for emotion classification had all reported to have no physical abnormalities or mental disorders and are thus fit and healthy for the experiments aside from one reported study which was granted permission to conduct on ASD subjects [117]. Other reports have evaluated their understanding of emotion labels before partaking any experiment as most of the participants would need to evaluate their emotions using Self-Assessment Manikin (SAM) after each trial. The studies also reported that the participants had sufficient educational backgrounds and therefore can justify their emotions when questioned on their current mental state. Many of the studies were conducted on university grounds with permission since the research of emotion classification was conducted by university-based academicians, and therefore, the population of the participants was mostly from university students.

Many of these reported studies only focused on the feature extractions from their EEG experiments or from SAM evaluations on valence, arousal, and dominance and presented their classification results at the end. Based on the current findings, no studies were found that conducted specifically differentiating the differences between male and female emotional responses or classifications. To have a reliable classification result, such studies should be conducted with at least 10 participants to have statistically meaningful results.

## 5. Discussion

One of the issues that emerged from this review is that there is a lack of studies conducted for virtual reality-based emotion classification where the immersive experience of the virtual reality could possibly evoke greater emotional responses over the traditional stimuli presented through computer monitors or audible speakers since virtual reality combines senses such as sight, hearing, and sense of “being there” immersively. There is currently no openly available database for VR-based emotion classification, where the stimuli have been validated for virtual reality usage in emotional responses. Many of the research have had to self-design their own emotional stimuli. Furthermore, there are inconsistencies in terms of the duration of the stimulus presented for the participants, especially in virtual reality where the emotion fluctuates greatly depending on the duration and content of the stimulus presented. Therefore, to keep the fluctuations of the emotions as minimal as possible as well as being direct to the intended emotional response, the length of the stimulus presented should be kept between 15 and 20 seconds. The reason behind this selected duration was that there is ample amount of time for the participants to explore the virtual reality environment to get oneself associated and stimulated enough that there are emotional responses received as feedback from the stimuli presented.

In recent developments for virtual reality, there are many available products in the market used for entertainment purposes with the majority of the products intended for gaming experiences such as Oculus Rift, HTC Vive, Playstation VR, and many other upcoming products. However, these products might be costly and overburdened with requirements such as the need for a workstation capable of handling virtual reality rendering environments or a console-specific device. Current smartphones have built-in inertial sensors such as gyroscope and accelerometers to measure direction and movement speed. Furthermore, this small and compact device has enough computational power to run virtual reality content provided with a VR headset and a set of earphones. The package for building a virtual reality environment is available using System Development Kits (SDKs) such as Unity3D which can be exported to multiple platforms making it versatile for deployments across many devices.

With regard to versatility, various machine learning algorithms are currently available for use in different applications, and these algorithms can achieve complex calculations with minimal time wasted thanks to the technological advancements in computing as well as efficient utilization of algorithmic procedures [151]. However, there is no evidence of a single algorithm that can best the rest and this makes it difficult for algorithm selection when preparing for emotion classification tasks. Furthermore, with regard to versatility, there needs to be a trained model for machine learning algorithms that can be used for commercial deployment or benchmarking for future emotion classifications. Therefore, intersubject variability (also known as subject-dependent, studies across subjects, or leave-one-out in some other studies) is a concept that should be followed as this method generalizes the emotion classification task over the overall population and has a high impact value due to the nonrequirement of retraining the classification model for every single new user.

The collection of brainwave signals varies differently depending on the quality or sensitivity of the electrodes when attempting to collect the brainwave signals. Furthermore, the collection of brainwave signals depends on the number of electrodes and its placements around the scalp which should conform to the 10–20 international EEG standards. There needs to be a standardized measuring tool for the collection of EEG signals, and the large variances of products of wearable EEG headsets would produce varying results depending on the handlings of the user. It is suggested that standardization for the collection of the brainwave signals be accomplished using a low-cost wearable EEG headset since it is easily accessible by the research community. While previous studies have reported that the emotional experiences are stored within the temporal region of the brain, current evidence suggests that emotional responses may also be influenced by different regions of the brain such as the frontal and parietal regions. Furthermore, the association of brainwave bands from both the lower and higher frequencies can actually improve the emotional classification accuracy. Additionally, the optimal selection of the electrodes as learning features should also be considered since many of the EEG devices have different numbers of electrodes and placements, and hence, the number and selection of electrode positions should be explored systematically in order to verify how it affects the emotion classification task.

## 6. Conclusions

In this review, we have presented the analysis of emotion classification studies from 2016–2019 that propose novel methods for emotion recognition using EEG signals. The review also suggests a different approach towards emotion classification using VR as the emotional stimuli presentation platform and the need for developing a new database based on VR stimuli. We hope that this paper has provided a useful critical review update on the current research work in EEG-based emotion classification and that the future opportunities for research in this area would serve as a platform for new researchers venturing into this line of research.

## Figures and Tables

**Table 1:** Market available for EEG headset between low and middle cost.

Product tier	Products	Channel positions	Sampling rate	Electrodes	Cost
Low-cost range (USD99-USD 1,000)	Emotiv EPOC+	AF3, F7, F3, FC5, T7, P7, O1, O2, P8, T8, FC6, F4, F8, AF4	32 Hz–64 Hz	14	USD 799.00
	NeuroSky MindWave	FP1	512 Hz	1	USD 99.00
	Ultracortex “Mark IV” EEG headset	FP2, FP1, C4, C3, P8, P7, O2, O1	128 Hz	8–16	USD 349.99
	Interaxon Muse	AF7, AF8, TP9, TP10	256 Hz	4	USD 250.00

Middle-cost range (USD 1,000-USD 25,000)	B-Alert X Series	Fz, F3, F4, Cz, C3, C4, P3, P4, Poz	256 Hz	10	(Undisclosed)
	ANT-Neuro eego rt	AF7, AF3, AF4, AF8, F5, F1, F2, F6, FT7, FC3, FCZ, FC4, FT8, C5, C1, C2, C6, TP7, CP3, CPz, CP4, TP8, P5, P1, P2, P6, PO7, PO5, PO3, PO4, PO6, PO8	2048 Hz	64	(Undisclosed)

**Table 2:** EEG signals and its frequency bands.

Band name	Frequency band (Hz)	Functions
Delta	<4	Usually associated with the unconscious mind and occurs in deep sleep
Theta	4–7	Usually associated with the subconscious mind and occurs in sleeping and dreaming
Alpha	8–15	Usually associated with a relaxed mental state yet aware and are correlated with brain activation
Beta	16–31	Usually associated with active mind state and occurs during intense focused mental activity
Gamma	>32	Usually associated with intense brain activity

**Table 3:** Publicly available datasets for emotion stimulus and emotion recognition with different methods of collection for neurophysiological signals.

Item No.	Dataset	Description
1	DEAP	“Dataset for Emotion Analysis using Physiological and Video Signals” is an open-source dataset to analyze human affective states. The dataset consists of 32 recorded participants watching 40 music video clips with a certain level of stimuli evaluated
2	IADS	“The International Affective Digital Sounds” system is a collection of digital sounds that is used to stimulate emotional responses through acoustics and is used in investigations of emotion and attention of an individual
	IAPS	“The International Affective Picture” system is a collection of the emotionally evocative picture that is used to stimulate emotional responses to investigate the emotion and attention of an individual
4	DREAMER	A dataset that has collected 23 participants with signals from EEG and ECG using audio-visual stimuli responses. The access of this dataset is restricted and can be requested upon filling a request form to the owner
5	ASCERTAIN	A “database for implicit personality and affect recognition” that collects signals from EEG, ECG, GSR, and facial activities from 58 individuals using 36 movie clips with an average length of 80 seconds
6	SEED	The “SJTU Emotion EEG Dataset” is a collection of EEG signals collected from 15 individuals watching 15 movie clips and measures the positive, negative, and neutral emotions
7	SEED-IV	An extension of the SEED dataset that now specifically targets the labels of the emotion specifically, happy, sad, fear, and neutral with an additional eye tracking feature added into the collection data inclusive of the EEG signal

**Table 4:** Comparison of stimuli used for the evocation of emotions, length of stimulus video, and emotion class evaluation.

Research author	Stimuli	Dataset	Clip length	Emotion classes
[92]	Music	IADS (4 songs)	60 sec per clip	Pleasant, happy, frightened, angry
[93]	Music	Self-Designed (40 songs)	—	Happy, angry, afraid, sad
[94]	Music	Self-Designed (301 songs collected from different albums)	30 sec per clip	Happy, angry, sad, peaceful
[95]	Music	Self-Designed (1080 songs)	—	Anger, sadness, happiness, boredom, calm, relaxation, nervousness, pleased, and peace
[96]	Music	Self-Designed (3552 songs from Baidu)	—	Contentment, depression, exuberance
[97]	Music	1000 songs from MediaEval	45 sec per clip	Pleasing, angry, sad, relaxing
[98]	Music	Self-Designed (25 songs + Healing4Happiness dataset)	247.55 sec	Valence, arousal
[99]	Music + picture	IAPS, Quran Verse, Self-Designed (Musicovery, AMG, Last.fm)	60 sec per clip	Happy, fear, sad, calm
[100]	Music videos	DEAP (40 music videos)	60 sec per clip	Valence, arousal, dominance, liking
[101]	Music videos	DEAP (40 music videos)	—	Valence, arousal
[102]	Music videos	DEAP (40 music videos)	60 sec per clip	Valence, arousal
[103]	Music videos	DEAP (40 music videos)	60 sec per clip	—
[104]	Music videos	DEAP (40 music videos)	60 sec per clip	Valence, arousal
[105]	Music videos	DEAP (40 music videos)	60 sec per clip	Valence, arousal, dominance
[106]	Video clips	Self-Designed (12 video clips)	150-sec per clip	Happy, fear, sad, relax
[107]	Video clips	DECAF (36 video clips) [108]	51–128 sec per clip	Valence, arousal
[109]	Video clips	Self-designed (15 video clips)	120–240 sec per clip	Happy, sad, fear, disgust, neutral
[110]	Video clips	SEED (15 video clips), DREAMER (18 video clips)	SEED (240 sec per clip), DREAMER (65–393 sec per clip)	Negative, positive, and neutral (SEED). Amusement, excitement, happiness, calmness, anger, disgust, fear, sadness, and surprise (DREAMER)
[111]	Video clips	SEED (15 video clips)	240 sec per clip	Positive, neutral, negative
[112]	Video clips	Self-Designed (20 video clips)	120 sec per clip	Valence, arousal
[113]	VR	Self-Designed (4 scenes)	—	Arousal and valence
[114]	VR	AVRS (8 scenes)	80 sec per scene	Happy, sad, fear, relaxation, disgust, rage
[115]	VR	Self-Designed (2 video clips)	475 sec + 820 sec clip	Horror, empathy
[116]	VR	Self-Designed (5 scenes)	60 sec per scene	Happy, relaxed, depressed, distressed, fear
[117]	VR	Self-Designed (1 scene)	—	Engagement, enjoyment, boredom, frustration, workload
[118]	VR	Self-Designed (1 scene that changes colour intensity)	—	Anguish, tenderness
[114]	VR	AVRS (4 scenes)	—	Happy, fear, Peace, disgust, sadness
[119]	VR	NAPS (Nencki Affective Picture System) (20 pictures)	15 sec per picture	Happy, fear
[120]	VR	Self-Designed (1 scene)	90 sec per clip	Fear

**Table 5:** Common EEG headset recordings, placements, and types of brainwave recordings.

Research author	EEG headset model used	Brief description of electrode placements	Frequency bands recorded
[102]	BioSemi ActiveTwo	Prefrontal, prefrontal-frontal, frontal, frontal-central, temporal, central, central-parietal, parietal, parietal-occipital, occipital	Theta, alpha, lower-beta, upper-beta, gamma
[130]	NeuroSky MindWave	Prefrontal	Delta, theta, low-alpha, high-alpha, low-beta, high-beta, low-gamma, mid-gamma
[120]	actiChamp	Frontal, central, parietal, occipital	Delta, theta, alpha, beta, gamma
[109]	AgCl Electrode Cap	—	Delta, theta, alpha, beta, gamma
[103]	BioSemi ActiveTwo	Frontal	Delta, theta, alpha, beta, gamma
[104]	BioSemi ActiveTwo	Prefrontal, prefrontal-frontal, frontal, frontal-central, temporal, central, central-parietal, parietal, parietal-occipital, occipital	Delta, theta, alpha, beta, gamma
[105]	BioSemi ActiveTwo	Prefrontal, prefrontal-frontal, frontal, frontal-central, temporal, central, central-parietal, parietal, parietal-occipital, occipital	Delta, theta, alpha, beta, gamma
[117]	Emotiv EPOC+	Prefrontal-frontal, frontal, frontal-central, temporal, parietal, occipital, frontal-central	Delta, theta, alpha, beta, gamma
[58]	Muse	Temporal-parietal, prefrontal-frontal	Delta, theta, alpha, beta, gamma
[107]	NeuroSky MindWave	Prefrontal	Delta, theta, alpha, beta, gamma
[119]	Emotiv EPOC+	Prefrontal-frontal, frontal, frontal-central, temporal, parietal, occipital, frontal-central	Alpha, low-beta, high-beta, gamma, theta
[101]	BioSemi ActiveTwo	Prefrontal, prefrontal-frontal, frontal, frontal-central, temporal, central, central-parietal, parietal, parietal-occipital, occipital	Alpha, beta
[112]	Ag/AgCK Subtered Ring Electrodes	Fp1, T3, F7, O1, T4, Fp2, C3, T5, F3, P3, T6, P4, O2, F4, F8	—
[113]	B-Alert X10	Frontal, central, parietal	—
[100]	BioSemi ActiveTwo	Prefrontal, prefrontal-frontal, frontal, frontal-central, temporal, central, central-parietal, parietal, parietal-occipital, occipital	—

**Table 6:** Comparison of classifiers used for emotion classification and its performance.

Research author	Classifiers	Best performance achieved	Intersubject or Intrasubject
[110]	Dynamical graph convolutional neural network	90.40%	Intrasubject and intersubject
[140]	Support vector machine	80.76%	Intrasubject and intersubject
[93]	Random forest, instance-based	98.20%	Intrasubject
[118]	Support vector machine	—	Intrasubject
[99]	Multilayer perceptron	76.81%	Intrasubject
[117]	K-nearest neighbor	95.00%	Intersubject
[92]	Support vector machine	73.10%	Intersubject
[104]	Support vector machine, K-nearest neighbor, convolutional neural network, deep neural network	82.81%	Intersubject
[141]	Support vector machine	81.33%	Intersubject
[102]	Support vector machine, convolutional neural network	81.14%	Intersubject
[103]	Gradient boosting decision tree	75.18%	Intersubject
[113]	Support vector machine	70.00%	Intersubject
[100]	Support vector machine	70.52%	Intersubject
[107]	Support vector machine, naïve Bayes	61.00%	Intersubject
[142]	Support vector machine	57.00%	Intersubject
[94]	Support vector machine, K-nearest neighbor	—	Intersubject
[111]	Support vector machine, K-nearest neighbor	98.37%	—
[143]	Convolutional neural network	97.69%	—
[144]	Support vector machine, backpropagation neural network, late fusion method	92.23%	—
[145]	Fisherface	91.00%	—
[93]	Haar, Fisherface	91.00%	—
[106]	Extreme learning machine	87.10%	—
[112]	K-nearest neighbor, support vector machine, multilayer perceptron	86.27%	—
[97]	Support vector machine, K-nearest neighbor, fuzzy networks, Bayes, linear discriminant analysis	83.00%	—
[105]	Naïve Bayes, support vector machine, K-means, hierarchical clustering	78.06%	—
[130]	Support vector machine, naïve Bayes, multilayer perceptron	71.42%	—
[95]	Gaussian process	71.30%	—
[96]	Naïve Bayes	68.00%	—

**Table 7:** Reported number of participants used to conduct emotion classification.

Author	Emotion classes	Participants	Male	Female	Mean age ± SD
[114]	Happy, sad, fear, relaxation, disgust, rage	100	57	43	—
[113]	Arousal and valence (4 quadrants)	60	16	44	28.9 ± 5.44
[149]	Valence, arousal	58 (ASCERTAIN)	37	21	30
[107]	Valence, arousal	58 (ASCERTAIN)	37	21	30
[112]	Valence, arousal (high and low)	40	20	20	26.13 ± 2.79
[110]	Negative, positive, and neutral (SEED). Amusement, excitement, happiness, calmness, anger, disgust, fear, sadness, and surprise (DREAMER)	15 (SEED), 23 (DREAMER)	21	17	26.6 ± 2.7
[115]	Horror = (fear, anxiety, disgust, surprise, tension), empathy = (happiness, sadness, love, being touched, compassion, distressing, disappointment)	38	19	19	—
[100]	Valence, arousal, dominance, liking	32 (DEAP)	16	16	26.9
[101]	Valence, arousal (high and low)	32 (DEAP)	16	16	26.9
[102]	Valence, arousal	32 (DEAP)	16	16	26.9
[103]	—	32 (DEAP)	16	16	26.9
[104]	Valence, arousal (2 class)	32 (DEAP)	16	16	26.9
[105]	Valence, arousal, dominance	32 (DEAP)	16	16	26.9
[114]	Happy, fear, peace, disgust, sadness	13 (watching video materials), 18 (VR materials)	13	18	—
[130]	Stress level (low and high)	28	19	9	27.5
[98]	Valence, arousal (high and low)	25	—	—	—
[120]	Fear	22	14	8	—
[106]	Happy, fear, sad, relax	20	—	—	—
[117]	Engagement, enjoyment, boredom, frustration, workload	20	19	1	15.29
[109]	Happy, sad, fear, disgust, neutral	16	6	10	23.27 ± 2.37
[118]	Anguish, tenderness	16	—	—	—
[111]	Positive, neutral, negative	15 (SEED)	7	8	—
[99]	Happy, fear, sad, calm	13	8	5	—
[141]	Happy, relaxed, depressed, distressed, fear	10	10	—	21
[119]	Happy, fear	6	5	1	26.67 ± 1.11
[92]	Pleasant, happy, frightened, angry	5	4	1	—

## Data Availability

No data are made available for this work.

## References

[1] Mert A., Akan A. (2018). Emotion recognition from EEG signals by using multivariate empirical mode decomposition. *Pattern Analysis and Applications*.

[2] Bradley M. M., Lang P. J. (1994). Measuring emotion: the self-assessment manikin and the semantic differential. *Journal of Behavior Therapy and Experimental Psychiatry*.

[3] Morris J. (1995). Observations: SAM: the Self-Assessment Manikin; an efficient cross-cultural measurement of emotional response. *Journal of Advertising Research*.

[4] Hayashi E. C. S., Posada J. E. G., Maike V. R. M. L., Baranauskas M. C. C. Exploring new formats of the Self-Assessment Manikin in the design with children.

[5] Casson A. J. (2019). Wearable EEG and beyond. *Biomedical Engineering Letters*.

[6] Chen Y.-H., de Beeck M., Vanderheyden L. (2014). Soft, comfortable polymer dry electrodes for high quality ECG and EEG recording. *Sensors*.

[7] Boon G., Aricò P., Borghini G., Sciaraffa N., Di Florio A., Babiloni F. (2019). The dry revolution: evaluation of three different eeg dry electrode types in terms of signal spectral features, mental states classification and usability. *Sensors (Switzerland)*.

[8] Jeon S., Chien J., Song C., Hong J. (2018). A preliminary study on precision image guidance for electrode placement in an EEG study. *Brain Topography*.

[9] Kakisaka Y., Alkawadri R., Wang Z. I. (2013). Sensitivity of scalp 10–20 EEG and magnetoencephalography. *Epileptic Disorders*.

[10] Burgess M., Kumar A., J V. M. (2012). Analysis of EEG using 10:20 electrode system. *International Journal of Innovative Research in Science, Engineering and Technology*.

[11] Bigirimana A. D., Siddique N., Coyle D. A hybrid ICA-wavelet transform for automated artefact removal in EEG-based emotion recognition.

[12] Bogacz R., Markowska-Kaczmar U., Kozik A. Blinking artefact recognition in EEG signal using artificial neural network.

[13] O’Regan S., Faul S., Marnane W. (2013). Automatic detection of EEG artefacts arising from head movements using EEG and gyroscope signals. *Medical Engineering and Physics*.

[14] Romo-Vazquez R., Ranta R., Louis-Dorr V., Maquin D. EEG ocular artefacts and noise removal.

[15] Islam M. K., Rastegarnia A., Yang Z. (2016). Methods for artifact detection and removal from scalp EEG: a review. *Neurophysiologie Clinique/Clinical Neurophysiology*.

[16] Janani A. S., Grummett T. S., Lewis T. W. (2018). Improved artefact removal from EEG using Canonical Correlation Analysis and spectral slope. *Journal of Neuroscience Methods*.

[17] Pope X., Bian G. B., Tian Z. (2019). Removal of artifacts from EEG signals: a review. *Sensors (Switzerland)*.

[18] Suja Priyadharsini S., Edward Rajan S., Femilin Sheniha S. (2016). A novel approach for the elimination of artefacts from EEG signals employing an improved Artificial Immune System algorithm. *Journal of Experimental & Theoretical Artificial Intelligence*.

[19] Szentkirályi A., Wong K. K. H., Grunstein R. R., D’Rozario A. L., Kim J. W. (2017). Performance of an automated algorithm to process artefacts for quantitative EEG analysis during a simultaneous driving simulator performance task. *International Journal of Psychophysiology*.

[20] Tandle A., Jog N., D’cunha P., Chheta M. (2016). Classification of artefacts in EEG signal recordings and EOG artefact removal using EOG subtraction. *Communications on Applied Electronics*.

[21] Murugappan M., Murugappan S. Human emotion recognition through short time Electroencephalogram (EEG) signals using Fast Fourier Transform (FFT).

[22] Alarcao S. M., Fonseca M. J. (2019). Emotions recognition using EEG signals: a survey. *IEEE Transactions on Affective Computing*.

[23] Panksepp J. (2004). *Affective Neuroscience: The Foundations of Human and Animal Emotions*.

[24] Penner A. E., Stoddard J. (2018). Clinical affective neuroscience. *Journal of the American Academy of Child & Adolescent Psychiatry*.

[25] Pessoa L. (2018). Understanding emotion with brain networks. *Current Opinion in Behavioral Sciences*.

[26] Ekman P., Friesen W. V. (1971). Constants across cultures in the face and emotion. *Journal of Personality and Social Psychology*.

[27] De Gelder B. (2009). Why bodies? Twelve reasons for including bodily expressions in affective neuroscience. *Philosophical Transactions of the Royal Society B: Biological Sciences*.

[28] Plaza-del-Arco F. M., Martín-Valdivia M. T., Ureña-López L. A., Mitkov R. (2020). Improved emotion recognition in Spanish social media through incorporation of lexical knowledge. *Future Generation Computer Systems*.

[29] Kumar J., Kumar J. A. (2015). Machine learning approach to classify emotions using GSR. *Advanced Research in Electrical and Electronic Engineering*.

[30] Ali M., Mosa A. H., Al Machot F., Kyamakya K. (2018). Emotion recognition involving physiological and speech signals: a comprehensive review. *Recent Advances in Nonlinear Dynamics and Synchronization*.

[31] Hockenbury D. H., Hockenbury S. E. (2010). *Discovering Psychology*.

[32] Mauss I. B., Robinson M. D. (2009). Measures of emotion: a review. *Cognition & Emotion*.

[33] Fox E. (2008). *Emotion Science Cognitive and Neuroscientific Approaches to Understanding Human Emotions*.

[34] Ekman P. (1992). Are there basic emotions?. *Psychological Review*.

[35] Plutchik R. (2001). The nature of emotions. *American Scientist*.

[36] Izard C. E. (2007). Basic emotions, natural kinds, emotion schemas, and a new paradigm. *Perspectives on Psychological Science*.

[37] Izard C. E. (2009). Emotion theory and research: highlights, unanswered questions, and emerging issues. *Annual Review of Psychology*.

[38] Lang P. J. (1995). The emotion probe: studies of motivation and attention. *American Psychologist*.

[39] Mehrabian A. (1997). Comparison of the PAD and PANAS as models for describing emotions and for differentiating anxiety from depression. *Journal of Psychopathology and Behavioral Assessment*.

[40] Osuna E., Rodríguez L., Gutierrez-garcia J. O., Luis A., Osuna E., Rodr L. (2020). Development of computational models of Emotions : a software engineering perspective. *Cognitive Systems Research*.

[41] Hassouneh A., Mutawa A. M., Murugappan M. (2020). Development of a real-time emotion recognition system using facial expressions and EEG based on machine learning and deep neural network methods. *Informatics in Medicine Unlocked*.

[42] Balducci F., Grana C., Cucchiara R. (2017). Affective level design for a role-playing videogame evaluated by a brain-computer interface and machine learning methods. *The Visual Computer*.

[43] Su Z., Xu X., Jiawei D., Lu W. Intelligent wheelchair control system based on BCI and the image display of EEG.

[44] Campbell A., Choudhury T., Hu S. NeuroPhone: brain-mobile phone interface using a wireless EEG headset.

[45] Bright D., Nair A., Salvekar D., Bhisikar S. EEG-based brain controlled prosthetic arm.

[46] Demirel C., Kandemir H., Kose H. Controlling a robot with extraocular muscles using EEG device.

[47] Liu Y., Ding Y., Li C. (2020). Multi-channel EEG-based emotion recognition via a multi-level features guided capsule network. *Computers in Biology and Medicine*.

[48] Ahern G. L., Schwartz G. E. (1985). Differential lateralization for positive and negative emotion in the human brain: EEG spectral analysis. *Neuropsychologia*.

[49] Gunes H., Piccardi M. (2007). Bi-modal emotion recognition from expressive face and body gestures. *Journal of Network and Computer Applications*.

[50] Jenke R., Peer A., Buss M. (2014). Feature extraction and selection for emotion recognition from EEG. *IEEE Transactions on Affective Computing*.

[51] Blackford J. U., Pine D. S. (2012). Neural substrates of childhood anxiety disorders. *Child and Adolescent Psychiatric Clinics of North America*.

[52] Goosens K. A., Maren S. (2002). Long-term potentiation as a substrate for memory: evidence from studies of amygdaloid plasticity and pavlovian fear conditioning. *Hippocampus*.

[53] Turner M. R., Maren S., Phan K. L., Liberzon I. (2013). The contextual brain: implications for fear conditioning, extinction and psychopathology. *Nature Reviews Neuroscience*.

[54] Herwig U., Satrapi P., Schönfeldt-Lecuona C. (2003). Using the international 10–20 EEG system for positioning of transcranial magnetic stimulation. *Brain Topography*.

[55] Homan R. W., Herman J., Purdy P. (1987). Cerebral location of international 10–20 system electrode placement. *Electroencephalography and Clinical Neurophysiology*.

[56] Rojas G. M., Alvarez C., Montoya C. E., de la Iglesia-Vayá M., Cisternas J. E., Gálvez M. (2018). Study of resting-state functional connectivity networks using EEG electrodes position as seed. *Frontiers in Neuroscience*.

[57] Blanco J. A., Vanleer A. C., Calibo T. K., Firebaugh S. L. (2019). Single-trial cognitive stress classification using portable wireless electroencephalography. *Sensors (Switzerland)*.

[58] Abujelala M., Sharma A., Abellanoza C., Makedon F. Brain-EE: brain enjoyment evaluation using commercial EEG headband.

[59] Chew L. H., Teo J., Mountstephens J. Aesthetic preference recognition of 3D shapes using EEG. *Cognitive Neurodynamics*.

[60] Mountstephens G., Yamada T. (2018). Pediatric clinical neurophysiology. *Atlas of Artifacts in Clinical Neurophysiology*.

[61] Miller C. (2015). Review of handbook of EEG interpretation. *The Neurodiagnostic Journal*.

[62] Obeid I., Picone J. (2016). The temple university hospital EEG data corpus. *Frontiers in Neuroscience*.

[63] Aldridge A., Barnes E., Bethel C. L. Accessible electroencephalograms (EEGs): A comparative review with openbci’s ultracortex mark IV headset.

[64] Bialas P., Milanowski P. A high frequency steady-state visually evoked potential based brain computer interface using consumer-grade EEG headset.

[65] Wang Y., Wang Z., Clifford W., Markham C., Ward T. E., Deegan C. Validation of low-cost wireless EEG system for measuring event-related potentials.

[66] Sridhar S., Ramachandraiah U., Sathish E., Muthukumaran G., Prasad P. R. Identification of eye blink artifacts using wireless EEG headset for brain computer interface system.

[67] Ahmad M., Aqil M. Implementation of nonlinear classifiers for adaptive autoregressive EEG features classification.

[68] Mheich A., Guilloton J., Houmani N. Monitoring visual sustained attention with a low-cost EEG headset.

[69] Tomonaga K., Wakamizu S., Kobayashi J. Experiments on classification of electroencephalography (EEG) signals in imagination of direction using a wireless portable EEG headset.

[70] Wakamizu S., Tomonaga K., Kobayashi J. Experiments on neural networks with different configurations for electroencephalography (EEG) signal pattern classifications in imagination of direction.

[71] Sarno R., Munawar M. N., Nugraha B. T. (2016). Real-time electroencephalography-based emotion recognition system. *International Review on Computers and Software (IRECOS)*.

[72] Thammasan N., Moriyama K., Fukui K.-i., Numao M. (2017). Familiarity effects in EEG-based emotion recognition. *Brain Informatics*.

[73] Zhuang N., Zeng Y., Tong L., Zhang C., Zhang H., Yan B. (2017). Emotion recognition from EEG signals using multidimensional information in EMD domain. *BioMed Research International*.

[74] Lee T. M. C., Liu H.-L., Chan C. C. H., Fang S.-Y., Gao J.-H. (2005). Neural activities associated with emotion recognition observed in men and women. *Molecular Psychiatry*.

[75] Zhu J.-Y., Zheng W.-L., Lu B.-L. (2015). Cross-subject and cross-gender emotion classification from EEG. *World Congress on Medical Physics and Biomedical Engineering*.

[76] Stanica I., Dascalu M. I., Bodea C. N., Bogdan Moldoveanu A. D. VR job interview simulator: where virtual reality meets artificial intelligence for education.

[77] Malandrakis N., Potamianos A., Evangelopoulos G., Zlatintsi A. (2011). *A Supervised Approach To Movie Emotion Tracking*.

[78] Ip H. H. S., Wong S. W. L., Chan D. F. Y. (2018). Enhance emotional and social adaptation skills for children with autism spectrum disorder: a virtual reality enabled approach. *Computers & Education*.

[79] Wong J. (1998). What is virtual reality?. *Virtual Reality Information Resources*.

[80] Sutherland I. E., Fluke C. J., Barnes D. G. (1965). The ultimate display. Multimedia: from wagner to virtual reality. http://arxiv.org/abs/1601.03459.

[81] Klein R. G., Sutherland I. E. A head-mounted three dimensional display.

[82] Milgram P., Kishimo F. (1994). A taxonomy of mixed reality. *IEICE Transactions on Information and Systems*.

[83] Pan Z., Cheok A. D., Yang H., Zhu J., Shi J. (2006). Virtual reality and mixed reality for virtual learning environments. *Computers & Graphics*.

[84] Mekni M., Lemieux A. (2014). Augmented reality: applications, challenges and future trends. *Applied Computational Science*.

[85] Billinghurst M., Clark A., Lee G. (2014). A survey of augmented reality foundations and trends R in human-computer interaction. *Human-Computer Interaction*.

[86] Martin S., Diaz G., Sancristobal E., Gil R., Castro M., Peire J. (2011). New technology trends in education: seven years of forecasts and convergence. *Computers & Education*.

[87] Yang Y., Wu Q. M. J., Zheng W.-L., Lu B.-L. (2018). EEG-based emotion recognition using hierarchical network with subnetwork nodes. *IEEE Transactions on Cognitive and Developmental Systems*.

[88] Beemster T. T., van Velzen J. M., van Bennekom C. A. M., Reneman M. F., Frings-Dresen M. H. W. (2019). Test-retest reliability, agreement and responsiveness of productivity loss (iPCQ-VR) and healthcare utilization (TiCP-VR) questionnaires for sick workers with chronic musculoskeletal pain. *Journal of Occupational Rehabilitation*.

[89] Liu X., Zhang J., Hou G., Wang Z. (2018). Virtual reality and its application in military. *IOP Conference Series: Earth and Environmental Science*.

[90] Mcintosh J., Rodgers M., Marques B., Cadle A. (2019). *The Use of VR for Creating Therapeutic Environments for the Health and Wellbeing of Military Personnel , Their Families and Their Communities*.

[91] Johnson-Glenberg M. (2018). Immersive VR and education: embodied design principles that include gesture and hand controls. *Frontiers Robotics AI*.

[92] Lan Z., Sourina O., Wang L., Liu Y. (2016). Real-time EEG-based emotion monitoring using stable features. *The Visual Computer*.

[93] Kumaran D. S., Ragavendar S. Y., Aung A., Wai P. (2018). *Using EEG-validated Music Emotion Recognition Techniques to Classify Multi-Genre Popular Music for Therapeutic Purposes*.

[94] Lin C., Liu M., Hsiung W., Jhang J. (2017). Music emotion recognition based on two-level support vector classification. *Proceedings-International Conference on Machine Learning and Cybernetics*.

[95] Chen S. H., Lee Y. S., Hsieh W. C., Wang J. C. Music emotion recognition using deep Gaussian process.

[96] An Y., Sun S., Wang S. Naive Bayes classifiers for music emotion classification based on lyrics.

[97] Bai J., Luo K., Peng J. Music emotions recognition by cognitive classification methodologies.

[98] Nawaz R., Nisar H., Yap V. V. Recognition of useful music for emotion enhancement based on dimensional model.

[99] Al-Galal S. A. Y., Alshaikhli I. F. T., Rahman A. W. B. A., Dzulkifli M. A. EEG-based emotion recognition while listening to quran recitation compared with relaxing music using valence-arousal model.

[100] Shahnaz C., Masud S. B., Hasan S. M. S. Emotion recognition based on wavelet analysis of Empirical Mode Decomposed EEG signals responsive to music videos.

[101] Byun S. W., Lee S. P., Han H. S. Feature selection and comparison for the emotion recognition according to music listening.

[102] Xu J., Ren F., Bao Y. EEG emotion classification based on baseline strategy.

[103] Wu S., Xu X., Shu L., Hu B. Estimation of valence of emotion using two frontal EEG channels.

[104] Ullah H., Uzair M., Mahmood A., Ullah M., Khan S. D., Cheikh F. A. (2019). Internal emotion classification using EEG signal with sparse discriminative ensemble. *IEEE Access*.

[105] Dabas H., Sethi C., Dua C., Dalawat M., Sethia D. Emotion classification using EEG signals.

[106] Krishna A. H., Sri A. B., Priyanka K. Y. V. S., Taran S., Bajaj V. (2019). Emotion classification using EEG signals based on tunable-Q wavelet transform. *IET Science, Measurement & Technology*.

[107] Subramanian R., Wache J., Abadi M. K., Vieriu R. L., Winkler S., Sebe N. (2018). Ascertain: emotion and personality recognition using commercial sensors. *IEEE Transactions on Affective Computing*.

[108] Abadi M. K., Subramanian R., Kia S. M., Avesani P., Patras I., Sebe N. (2015). DECAF: MEG-based multimodal database for decoding affective physiological responses. *IEEE Transactions on Affective Computing*.

[109] Li T. H., Liu W., Zheng W. L., Lu B. L. Classification of five emotions from EEG and eye movement signals: discrimination ability and stability over time.

[110] Song T., Zheng W., Song P., Cui Z. (2018). EEG emotion recognition using dynamical graph convolutional neural networks. *IEEE Transactions on Affective Computing*.

[111] Kimmatkar N. V., Babu V. B. Human emotion classification from brain EEG signal using multimodal approach of classifier.

[112] Zangeneh Soroush M., Maghooli K., Kamaledin Setarehdan S., Motie Nasrabadi A. (2018). Emotion classification through nonlinear EEG analysis using machine learning methods. *International Clinical Neuroscience Journal*.

[113] Marín-Morales J., Higuera-Trujillo J. L., Greco A. (2018). Affective computing in virtual reality: emotion recognition from brain and heartbeat dynamics using wearable sensors. *Scientific Reports*.

[114] Zhang W., Shu L., Xu X., Liao D. Affective virtual reality system (AVRS): design and ratings of affective VR scenes.

[115] Kim A., Chang M., Choi Y., Jeon S., Lee K. The effect of immersion on emotional responses to film viewing in a virtual environment.

[116] Hidaka K., Qin H., Kobayashi J. Preliminary test of affective virtual reality scenes with head mount display for emotion elicitation experiment.

[117] Fan J., Wade J. W., Key A. P., Warren Z. E., Sarkar N. (2018). EEG-based affect and workload recognition in a virtual driving environment for ASD intervention. *IEEE Transactions on Biomedical Engineering*.

[118] Lorenzetti V., Melo B., Basílio R. (2018). Emotion regulation using virtual environments and real-time fMRI neurofeedback. *Frontiers in Neurology*.

[119] Horvat M., Dobrinic M., Novosel M., Jercic P. Assessing emotional responses induced in virtual reality using a consumer eeg headset: a preliminary report.

[120] Guo K., Huang J., Yang Y., Xu X. Effect of virtual reality on fear emotion base on EEG signals analysis.

[121] Koelstra S., Muhl C., Soleymani M. (2012). DEAP: a database for emotion analysis; using physiological signals. *IEEE Transactions on Affective Computing*.

[122] Patras A., Valenza G., Citi L., Scilingo E. P. (2017). Arousal and valence recognition of affective sounds based on electrodermal activity. *IEEE Sensors Journal*.

[123] Soleymani M., Caro M. N., Schmidt E. M., Sha C. Y., Yang Y. H. 1000 songs for emotional analysis of music..

[124] Huo X. Q., Zheng W. L., Lu B. L. Driving fatigue detection with fusion of EEG and forehead EOG.

[125] Soleymani M., Asghari-Esfeden S., Pantic M., Fu Y. Continuous emotion detection using EEG signals and facial expressions.

[126] Zheng W. L., Lu B. L. (2017). A multimodal approach to estimating vigilance using EEG and forehead EOG. *Journal of Neural Engineering*.

[127] Katsigiannis S., Ramzan N. (2018). DREAMER: a database for emotion recognition through EEG and ecg signals from wireless low-cost off-the-shelf devices. *IEEE Journal of Biomedical and Health Informatics*.

[128] Constantinescu A. C., Wolters M., Moore A., MacPherson S. E. (2017). A cluster-based approach to selecting representative stimuli from the International Affective Picture System (IAPS) database. *Behavior Research Methods*.

[129] Marchewka A., Żurawski Ł., Jednoróg K., Grabowska A. (2014). The Nencki Affective Picture System (NAPS): introduction to a novel, standardized, wide-range, high-quality, realistic picture database. *Behavior Research Methods*.

[130] Saeed S. M. U., Anwar S. M., Majid M., Bhatti A. M. Psychological stress measurement using low cost single channel EEG headset.

[131] Jerritta S., Murugappan M., Nagarajan R., Wan K. Physiological signals based human emotion recognition: a review.

[132] Maaoul C., Pruski A. (2010). Emotion recognition through physiological signals for human-machine communication. *Cutting Edge Robotics*.

[133] Liu C., Rani P., Sarkar N. An empirical study of machine learning techniques for affect recognition in human-robot interaction.

[134] Rigas G., Katsis C. D., Ganiatsas G., Fotiadis D. I. (2007). *A User Independent, Biosignal Based, Emotion Recognition Method*.

[135] Zong C., Chetouani M. Hilbert-Huang transform based physiological signals analysis for emotion recognition.

[136] Li L., Chen J. H. Emotion recognition using physiological signals from multiple subjects.

[137] Haag A., Goronzy S., Schaich P., Williams J. (2004). Emotion recognition using bio-sensors: first steps towards an automatic system. *Lecture Notes in Computer Science*.

[138] Kim J., Andre E. (2008). Emotion recognition based on physiological changes in music listening. *IEEE Transactions on Pattern Analysis and Machine Intelligence*.

[139] Nasoz F., Alvarez K., Lisetti C. L., Finkelstein N. (2004). Emotion recognition from physiological signals using wireless sensors for presence technologies. *Cognition, Technology & Work*.

[140] Li Y., Zheng W., Zong Y., Cui Z., Zhang T. (2019). A Bi-hemisphere domain adversarial neural network model for EEG emotion recognition. *IEEE Transactions on Affective Computing*.

[141] Zhou K., Qin H., Kobayashi J. Preliminary test of affective virtual reality scenes with head mount display for emotion elicitation experiment.

[142] Soleymani M., Lichtenauer J., Pun T., Pantic M. (2012). A multimodal database for affect recognition and implicit tagging. *IEEE Transactions on Affective Computing*.

[143] Gilda S., Zafar H., Soni C., Waghurdekar K. Smart music player integrating facial emotion recognition and music mood recommendation.

[144] Shi W., Feng S. Research on music emotion classification based on lyrics and audio.

[145] Iyer A. V., Pasad V., Sankhe S. R., Prajapati K. Emotion based mood enhancing music recommendation.

[146] Lin Y. P., Jung T. P. (2017). Improving EEG-based emotion classification using conditional transfer learning. *Frontiers in Human Neuroscience*.

[147] Lin Y. P., Wang C. H., Jung T. P. (2010). EEG-based emotion recognition in music listening. *IEEE Transactions on Bio-Medical Engineering*.

[148] Rinderknecht M. D., Lambercy O., Gassert R. (2018). Enhancing simulations with intra-subject variability for improved psychophysical assessments. *PLoS One*.

[149] Yoon J. H., Kim J. H. (2018). Wavelet-based statistical noise detection and emotion classification method for improving multimodal emotion recognition. *Journal of IKEEE*.

[150] Liao D., Zhang W., Liang G. Arousal evaluation of VR affective scenes based on HR and SAM.

[151] Karydis T., Aguiar F., Foster S. L., Mershin A. Performance characterization of self-calibrating protocols for wearable EEG applications.

